# Pollution Source Apportionment and Water Quality Risk Evaluation of a Drinking Water Reservoir during Flood Seasons

**DOI:** 10.3390/ijerph18041873

**Published:** 2021-02-15

**Authors:** Guoshuai Qin, Jianwei Liu, Shiguo Xu, Ya Sun

**Affiliations:** Institution of Water and Environment Research, Dalian University of Technology, Dalian 116024, China; qgs1991@mail.dlut.edu.cn (G.Q.); sgxu@dlut.edu.cn (S.X.); yasun_dlut@163.com (Y.S.)

**Keywords:** pollution source, apportionment, reservoir, water quality, risk evaluation, flood season

## Abstract

Reservoirs play an important role in the urban water supply, yet reservoirs receive an influx of large amounts of pollutants from the upper watershed during flood seasons, causing a decline in water quality and threatening the water supply. Identifying major pollution sources and assessing water quality risks are important for the environmental protection of reservoirs. In this paper, the principal component/factor analysis-multiple linear regression (PCA/FA-MLR) model and Bayesian networks (BNs) are integrated to identify water pollution sources and assess the water quality risk in different precipitation conditions, which provides an effective framework for water quality management during flood seasons. The deterioration of the water quality of rivers in the flood season is found to be the main reason for the deterioration in the reservoir water quality. The nonpoint source pollution is the major pollution source of the reservoir, which contributes 53.20%, 48.41%, 72.69%, and 68.06% of the total nitrogen (TN), phosphorus (TP), fecal coliforms (F.coli), and turbidity (TUB), respectively. The risk of the water quality parameters exceeding the surface water standard under different hydrological conditions is assessed. The results show that the probability of the exceedance rate of TN, TP, and F.coli increases from 91.13%, 3.40%, and 3.34%, to 95.75%, 25.77%, and 12.76% as the monthly rainfall increases from ≤68.25 mm to >190.18 mm. The risk to the water quality of the Biliuhe River reservoir is found to increase with the rising rainfall intensity, the water quality risk at the inlet during the flood season is found to be much greater than that at the dam site, and the increasing trend of TP and turbidity is greater than that of TN and F.coli. The risk of five-day biochemical oxygen demand (BOD_5_) does not increase with increasing precipitation, indicating that it is less affected by nonpoint source pollution. The results of this study can provide a research basis for water environment management during flood seasons.

## 1. Introduction

The uneven distribution of water resources and water pollution problems pose great challenges to water resource management on a global scale [1,2,3]. Reservoirs play an important role in flood control and water supply, but rapid socio-economic development has led to a decline in reservoir water quality, which has a significant impact on water resource utilization [4,5]. For the regions influenced by the monsoon climate, runoff is mainly concentrated in the flood season, it is necessary to store water for multiple uses of water supply, power generation, irrigation, etc. The flood season is also a period with a high incidence of water pollution emergencies, when pollutants in the watershed are washed into surface water by storm runoff, leading to water quality degradation [6,7,8]. Water contamination during flood seasons has been widely reported around the world [9,10,11,12,13]. For drinking water reservoirs, storm runoff is often impounded during the flood season, resulting in large amounts of pollutants entering the reservoirs, which have great impacts on the reservoir water supply. Water quality in reservoirs during flood seasons is influenced by multiple factors. Complex pollution sources and highly fluctuating hydrological factors increase the uncertainty of water quality during flood seasons. By identifying the major sources of pollutants entering reservoirs during floods, and analyzing their characteristics driven by precipitation, we can develop effective water quality management measures.

The apportionment of the water pollution sources is the foundation of environmental management in regard to surface water ecosystem, and general pollution source analysis methods include qualitative identification, quantitative identification, and a combination of qualitative and quantitative analysis [14,15,16]. Qualitative identification is to identify the main influencing factors by analyzing the intrinsic relationships in monitoring data, through principal component analysis, cluster analysis, and other multivariate statistical methods [16,17,18]. In quantitative analysis, receptor models are often used to analyze the contribution of pollution sources to the receptor environment by analyzing the physicochemical characteristics of the sources and the receptor environment. The receptor models mainly include the chemical mass balance model (CMB), positive definite matrix factor decomposition model (PMF), and principal component/factor analysis-multiple linear regression (PCA/FA-MLR) model [15,19,20]. Isotope tracer techniques have also been widely employed to resolve pollution sources and their contributions towards an environmental impact [21,22]. In addition, numerical modeling based on pollutant characteristics has been utilized to simulate the output and transport processes of pollutants, to determine the pollution sources and their contributions [23]. In other cases, the combination of remote sensing and hydrological characteristics provides a new approach to calculate the annual load of pollution sources [24]. Among the above methods, the CMB model requires a complete spectrum of emission source components, which is difficult to ascertain in reality, and the isotope method is limited to some extent by its high equipment requirements and complex analysis process [25]. Numerical modeling requires a comprehensive understanding of the transportation and transformation mechanisms of the pollutants, as well as a large amount of data to support it [26]. In contrast, the PMF and PCA/FA-MLR models depend less on the source component spectrum and mainly use the variation of water quality parameters to analyze the potential pollution sources and their contributions [15,27]. However, the model requires researchers to judge the number of pollution sources and their types, which may cause bias in the pollution source analysis on account of the different perceptions of the researchers [28,29].

Risk is generally used to indicate the likelihood of an adverse impact event, and water quality risk is a quantitative description of the likelihood of the occurrence of water pollution based on objectivity, uncertainty, measurability, and dynamics, the consequences of which are relatively controllable. Due to data limitations and the dynamics of the environment, the quantitative evaluation of water quality risk is complicated and difficult. Water quality risk assessment models can be divided into mechanistic, statistical, fuzzy mathematical, grey system, and coupling models based on different theories [30,31,32,33,34]. The Bayesian networks (BNs) model, developed based on Bayesian theory, is a widely-employed risk analysis model [35]. It has been shown that Bayesian networks that are based on water environment change mechanisms and statistical theory have great potential for water environment risk analysis, which has obvious advantages for quantifying uncertainty and calculating marginal risk, conditional risk, and the joint risk of water pollution incidents. Water environment risk analysis can be conducted in the face of multiscale and interdisciplinary problems [36]. Bertone et al. [37] develop a risk assessment tool based on BNs, system dynamics (SDs), and participatory modeling for managing the water-related health risks associated with extreme events. Liang et al. [38] utilized Bayesian networks to study the contributions of nitrogen and phosphorus concentrations to chlorophyll-a in different lake waters. Goulding et al. [39] studied the impact of sewage leaks on public health under rainfall conditions, which proved the advantages of Bayesian networks for water environment and water ecological uncertainty analysis. Besides this, some researchers have combined Bayesian networks with mechanistic models to fully utilize the advantages of both statistical and mechanistic models for the analysis of the water quality risks in sudden water pollution events, the results of which have been well-applied in different situations [34,37,40].

In this paper, a drinking water reservoir in Northeastern China is selected for the study of pollution sources and water quality risk during flood seasons. We first analyze the water quality characteristics in general, then identify the main sources of pollutants in the reservoir during the flood season, and analyze the contributions of each pollution source to the key water quality parameters by PCA/FA-MLR models. On this basis, a Bayesian network model is established to analyze the risk of water quality exceedance during the flood season and propose recommendations for watershed environment management.

## 2. Materials and Methods

### 2.1. Study Area

**Figure 1:**
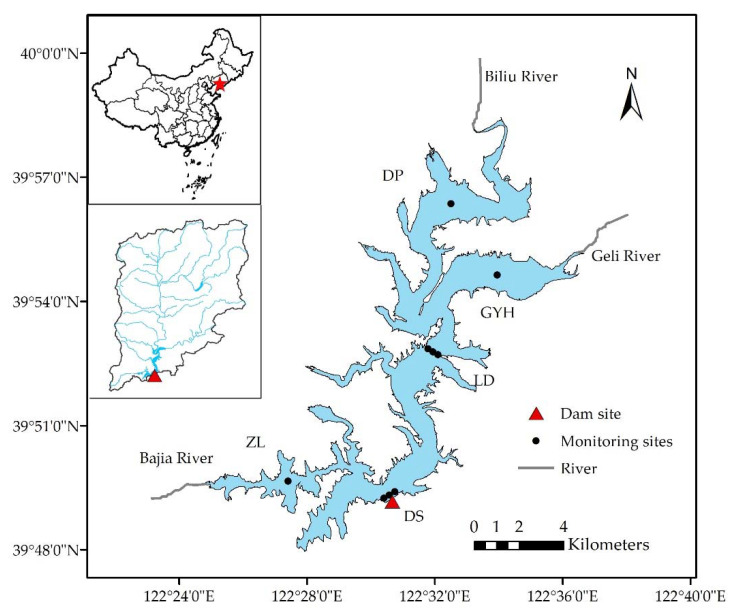
Geographical locations of water quality monitoring sites in the Biliuhe Reservoir, Liaoning Province, Northeast China.

The Biliuhe Reservoir (hereafter the BLH Reservoir) is a typical temperate reservoir located in the Liaoning Province, Northeast China. It has a surface area of 65.2 km^2^ with a mean and maximum water depth of 14.3 m and 31.0 m, respectively. The designed storage capacity of the reservoir is 9.34 × 10^8^ m^3^. The studied reservoir has been the most important water source for the city of Dalian since it was constructed in 1985. With an annual water supply of 3.0 × 10^8^ m³, it accounts for 80% of the domestic and industrial water supply to this city. Besides this, its water has multiple uses for flood control, irrigation, and electricity generation. The reservoir catchment reaches an area of about 2085 km^2^, with three main tributary rivers. The reservoir watershed has a temperate monsoon climate with a mean annual temperature of 10.6 °C, precipitation of 742 mm, and runoff of 6.14 × 10^8^ m³. The flood season (June–September) accounts for 75% and 82.4% of the total year’s precipitation and runoff, respectively. In the other half of the year, a period of freezing temperatures lasts from December to March. The primary land use types in the region are forests and farmland. The geographical locations of water quality monitoring sites in the study area are shown in Figure 1.

The hydrological data and water quality data for this study have been provided by the BLH Reservoir Bureau (BRB). The BRB has been monitoring the reservoir water quality regularly since 1988. The sampling frequency is once a month, and additional sampling will be conducted under special conditions such as floods. Samples are collected, transported, and tested by BRB according to national standards. In Figure 1, the notations DP, GYH, and ZL represent the entrance points of three main rivers, that is, the Biliuhe River (BLR), Geli River (GLR), and Bajia River (BJR). Liudian (LD), meanwhile, represents the central area of the reservoir, and DS represents the dam site (DS) area. A total of 12 parameters—pH, dissolved oxygen (DO), permanganate index (COD_Mn_), five-day biochemical oxygen demand (BOD_5_), ammonia (NH_3_-N), nitrate (NO_3_^−^-N), total nitrogen (TN), total phosphorus (TP), turbidity (TUB), fecal coliforms (F.coli), fluoride (F^−^), and chloride (Cl^−^)—have been selected for analysis in this study. The PCA/FA method requires all parameters to have the same timescale. As there are fewer recorded parameters and some missing values at the beginning of the data period, the data used for the PCA/FA-MLR model are those from 2006 to 2016. However, the Bayesian networks model is employed to analyze the water quality risk of individual indicators, so that all available data from 1988 to 2016 are included in the analysis.

### 2.2. Methods

#### 2.2.1. The PCA/FA-MLR Receptor Model

In this study, the PCA/FA method is used to reduce the data dimensionality and extract the most information from the original dataset based on the correlation of water quality variables [41,42]. Several new factors are generated to explain the variance of the whole dataset, and each component is identified as a pollution source [14,15]. Then, the receptor model combines the multiple linear regression model and the absolute principle component scores generated from a varimax rotated PCA to analyze the pollution contribution of each pollution source. This receptor model is one that was described in detail by Thurston and Spengler [43]. The source contribution of each component to the concentration of the variable can be described as follows:

(1) $$C_{i}=b_{0i}+\sum_{p=1}^{n}\left({\mathrm{APCS}}_{p}\cdot b_{pi}\right)$$

where *b*_0*i*_ is the constant term of the multiple regression for pollutant *i*, *b_pi_* is the multiple regression coefficients of the source *p* for pollutant *i*, and APCS*_p_* is the scaled value of the rotated factor *p* for the considered sample. The APCS*_p_* •*b_pi_* represents the contribution of source *p* to *C_i_*. In this study, SPSS 19.0 for Windows (SPSS Inc., Chicago, IL, USA) is used to perform the PCA/FA-MLR model.

#### 2.2.2. The Bayesian Networks Model

The study employs the Bayesian networks (BNs) model to analyze water quality risks in reservoirs. Bayesian networks have a flexible structure that can be adapted to the purpose of the study, which is a distinct advantage when dealing with interdisciplinary or complex problems [44]. A Bayesian network is a probabilistic inference model based on Bayesian theory and graph theory, consisting of a network structure G (Directed Acyclic Graph (DAG)), which qualitatively represents the dependencies between nodes, and a conditional probability table (CPT), which quantitatively represents the relationships between variables [35]. The joint probability distribution of BNs can be expressed as:

(2) $$P(X_{i})=\sum P(\pi_{X_{i}})P(X_{i}|\pi_{X_{i}})$$

(3) $$P(X_{1},X_{2},\cdots ,X_{n})=\prod P(X_{i}|\pi_{X_{i}})$$

where *P*(*π_Xi_*) is the prior probability, *P*(*X_i_*) is the node probability, *P*(*X_i_|π_Xi_*) is the conditional probability, and *P*(*X*_1_, *X*_2_,…, *X_n_*) is the joint probability. The Bayesian network inference is essentially a process that combines a priori information with new information to obtain a posteriori probabilities based on the Bayes Equation:

(4) $$P(\pi_{X_{i}}|X_{i})=\frac{P(\pi_{X_{i}})P(X_{i}|\pi_{X_{i}})}{\sum_{j=1}^{n}P(\pi_{X_{j}})P(X_{j}|\pi_{X_{j}})}$$

Bayesian networks have great advantages in the identification of relationships between the different influencing factors of complex systems. The Bayesian network modeling process mainly includes the following steps: determining the network structure, identifying network parameters, and drawing network inference [45,46]. In this study, the probabilistic inference was performed using the Bayesian network inference software, Genie2.0 (BayesFusion, LLC, Pittsburgh, PA, USA), a theoretical decision model for the graphical development environment of the building blocks, which can be easily utilized for Bayesian network inference due to its excellent operation mode and visual interface [47].

**Figure 2:**
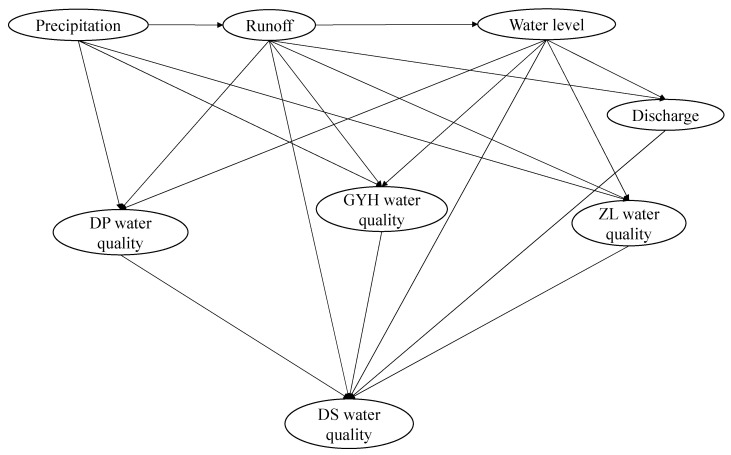
Bayesian network topology diagram.

In flood seasons, pollutant migration is mainly influenced by hydrological factors. This study focuses on the water quality risk posed by rainfall and runoff. Therefore, we take hydrological factors such as rainfall, runoff, water level, and reservoir discharge as input variables and assume that the emission of the pollution source is stable and changes little in different years. The water qualities at the inlet and dam site are taken as the output variables. For hydrological factors, the precipitation (P) is the most important hydrological elements for the hydrological cycle and material transportation, which is taken as the root node. The runoff (R), water level (W), and discharge (D) are directly or indirectly influenced by precipitation, which are taken as sub-nodes. Among them, the runoff and water level both determine the magnitude of reservoir discharge, while the discharge can also influence the water level. Because feedback loops must be avoided in Bayesian networks, it is assumed that the water level is mainly associated with the runoff in flood seasons. Further, the study constructs the relationship between hydrological parameters and water quality at the river entrance points (DPWQ, GYHWQ, ZLWQ) and the dam site (DSWQ) based on expert opinions. The relationship between hydrological factors and reservoir water quality is as follows: pollutants carried by storm runoff mainly affect the water quality at the river entrance area of the reservoir, which further influences the water quality at the dam site. The flood may also directly influence the water quality at the dam site in the form of the current density. Besides this, factors such as water level and discharge can also affect reservoir water quality to some extent. The water level can affect the thermal stratification and dilution storage of the reservoir, while discharge can affect the water quality by decreasing the hydraulic residence time. For the BLH Reservoir, the water level may affect the water quality at the river entrance points and dam site, while the discharge mainly affects the water quality at the dam site. The final topological structure of the Bayesian network is shown in Figure 2.

According to historical hydrological data, the frequencies of 75%, 50%, and 25% have been used to discretize the rainfall, runoff, water level, and discharge data in flood seasons. The discretization of water quality data is based on the environmental quality standard of surface water (GB3838–2002, Table 1), which can be divided into three states of S1 (type I), S2 (type II–III), and S3 (type IV–V) for most water quality parameters. The situation of total nitrogen is special, the concentration of TN is much higher than the standard of type III and even worse than that of type V in most cases. Therefore, three states of TN corresponding to the water quality of type I–III, type IV–V, and worse than type V. The discretization standards of hydrological parameters and main water quality parameters are shown in Table A1 and Table A2. According to the observed precipitation data of the BLH reservoir, the prior probabilities of precipitation in the S1 (<68.25 mm), S2 (68.25–119.46 mm), S3 (119.46–190.18 mm), and S4 (≥190.18 mm) states are 0.2500, 0.2727, 0.2500, and 0.2273, respectively. The conditional probabilities of other nodes are determined based on measured hydrological and water quality data from 1988–2016. Then, Bayesian networks are employed to determine the probability of exceedance (WQR|P) of water quality parameters at the entrance points of DP, GYH, and ZL, and the dam site (DS), under different rainfall conditions. Besides this, the Bayesian network model is also used to calculate the probability of exceeding the water quality standard at the dam site for different water quality states at the river entrance points (DSWQR|DBWQR, GYHWQR, ZLWQR).

## 3. Results

### 3.1. General Water Quality Characteristics in Different Seasons

As a major water source for the city of Dalian, the water quality of the BLH Reservoir should meet the requirements of surface water quality standard type III. The general water quality characteristics in non-flood and flood seasons of the BLH Reservoir are shown in Table 2. Among all of the water quality parameters, pH, DO, NH_3_-N, BOD_5_, and COD_Mn_ could meet standard type II most of the time. However, DO and BOD_5_ occasionally exceed the water quality standard. As for the nutrients, the concentration of TN exceeds the standard severely in both non-flood and flood seasons, with a concentration slightly lower in the flood period than in the non-flood period; the maximum value was 5.14 mg/L in the flood period, which is more than twice that specified by standard type V (2.0 mg/L). The mean concentration of TP was 0.021 mg/L during the flood seasons and TP exceeded standard type III occasionally. The TN and TP did not pass the significance test, but the highest value of TN and TP both occurred in the flood season. The BLH Reservoir is a phosphorus-limited reservoir, which is prone to eutrophication when the phosphorus concentration increases. The parameters of F.coli and turbidity, which are closely related to rainfall and runoff, were found to be significantly higher during flood seasons than that in non-flood seasons (Mann-Whitney U test, *p* < 0.01), especially for F.coli, which exceeded standard type III (10,000 A/L) during the flood seasons. There were no obvious changes in the concentrations of F^−^ and Cl^−^ between flood and non-flood periods, indicating that they are less affected by rainfall and runoff. From the statistical results, it was found that the general water quality during the flood season was worse than in the non-flood season, with the exceedance risk of TN, TP, F.coli, and BOD_5_. The excessive presentation of fecal coliform suggests the fecal pollution of the water body, which will have a great impact on the water supplied from the source. The increase in TN and TP will lead to a severe level of eutrophication and water quality degradation. The BOD_5_ indicates organic pollutants, which will reduce the level of dissolved oxygen in the water, produce odor, and affect the utilization of the water source. In the next step, it is necessary to analyze the pollution sources for those parameters that exceed the standard.

### 3.2. Pollution Source Apportionment during Flood Seasons

#### 3.2.1. Data Structure Determination and Source Identification Using PCA/FA

The Kaiser-Meyer-Olkin (KMO) and Bartlett’s sphericity tests were performed on the datasets before conducting the PCA/FA. The KMO value for the flood seasons was 0.688 and the Bartlett’s sphericity test value was 314.042 (*p* = 0.00 < 0.05), which indicated that the PCA/FA was effective in reducing the dimensionality of the water quality datasets. According to previous research, the absolute factor loading values of >0.75, 0.5–0.75, and 0.3–0.5 are considered to be ‘strong’, ‘moderate’, and ‘weak’, respectively. The larger the factor loading value of a water quality parameter, the greater the influence of that principal factor on the water quality [12]. In general, factors with initial eigenvalues greater than one were selected for analysis, but only 59.663% of the variance was explained. Therefore, an additional factor was added, and a total of 74.319% of the variance was explained, for four of the principal factors extracted. The calculated results of the factor analysis are shown in Table 3.

As shown in Table 3, factor 1 explained 25.853% of the total variance, with strong loading values for fecal coliform and turbidity, and moderate loading values for nitrate, total nitrogen, and total phosphorus. Considering the characteristics of the watershed, turbidity can be understood to reflect sediment erosion in the area, which is largely influenced by the flushing effects of rainfall and runoff. The fecal coliform mainly comes from the manure produced by livestock and poultry breeding, the nitrogen and phosphorus are associated with agricultural activities such as the application of fertilizer and manure [48]. Therefore, Factor 1 was identified as an agricultural nonpoint source of pollution driven by rainfall and runoff.

Factor 2 accounted for 19.07% of the total variance and had strong and positive loading values for COD_Mn_ and BOD_5_, a moderate loading value for F^−^, and weak loading values for NH_3_-N and TP. The parameters of COD_Mn_ and BOD_5_ indicated organic pollutants that are closely related to domestic and industrial wastewater discharges. Therefore, Factor 2 is identified as a source of rural and urban sewage discharge.

Factor 3 explained 15.21% of the total variance, with a strong loading value for Cl^−^, moderate loading values for NH_3_-N and NO_3_-N, and a weak loading value for TN. The concentration of chloride was relatively low in the reservoir. The chloride in the surface water is mainly influenced by the leaching of soil and rock, which may come from the groundwater input. Existing studies have shown that groundwater inputs are an important source of nitrogen for surface water ecosystems [20]. Therefore, Factor 3 could be identified as a groundwater pollution source.

Factor 4 accounted for 14.65% of the total variance and had strong and positive loading values for pH and DO. The DO in the surface water is mainly influenced by the reaeration rate and the microbial and chemical oxidation processes of organic and reducing compounds. The concentration of organic pollutants and reducing compounds in the BLH Reservoir was low, DO was closely related to meteorological factors, such as air temperature and wind, which can influence the reaeration rate. Therefore, Factor 4 could be identified as meteorological factors.

#### 3.2.2. Source Apportionment Using APCS–MLR Models

Once the identification of pollution sources in the region was complete, the contributions of the different pollution sources to different water quality parameters could be determined using an APCS–MLR receptor model. The calculation results are shown in Table 4. It can be seen from the table that meteorological factors contributed 77.41% and 82.10% to pH and DO, respectively, indicating that the pH and DO variations in the BLH Reservoir are mainly influenced by meteorological factors. The COD_Mn_ and BOD_5_, meanwhile, were greatly influenced by domestic and industrial sewage discharge sources, with contributions of 61.59% and 60.28%, respectively. The groundwater input contributed 52.98% to NH_3_-N. For the nutrients in the reservoir, an agricultural nonpoint pollution source was found to have contributed the most, with 53.20% and 48.41% for TN and TP, respectively. The F.coli and turbidity resulted mainly from agricultural nonpoint pollution sources, contributing 72.69% and 68.06%, respectively. Fluoride was influenced by sewage discharge sources, with a contribution of 49.45%, and chloride most was influenced by groundwater input, with a contribution of 61.31%. The results demonstrated that an agricultural nonpoint source was the main contributor to pollution during the flood seasons; this source contributed in the largest proportion to the water parameters that exceeded the standard. Besides this, other sources such as sewage discharge and groundwater pollution also need attention due to the visible contributions.

### 3.3. Water Quality Risk Evaluation during Flood Seasons

#### 3.3.1. Water Quality Risks Based on the Prior Bayesian Network

**Figure 3:**
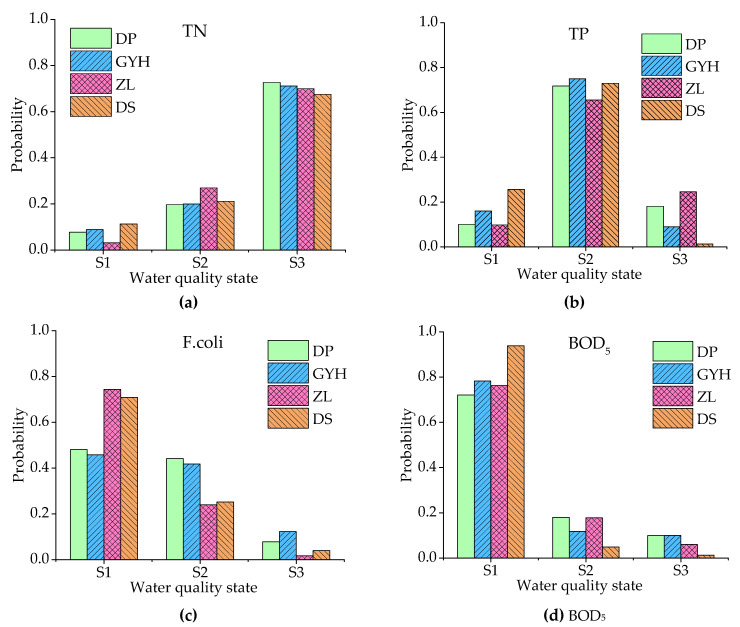
Water quality risk of different variables (**a**) total nitrogen (TN), (**b**) phosphorus (TP), (**c**) fecal coliforms (F.coli), and (**d**) five-day biochemical oxygen demand (BOD5) based on the prior Bayesian network.

According to the results of Section 3.1 and Section 3.2, TN, TP, F.coli, and BOD_5_ were the main water quality parameters with the risk for exceeding the standard in flood seasons. These four parameters were selected for further analysis. The probabilities of TN, TP, F.coli, and BOD_5_ in different water quality states, based on prior probabilities, are shown in Figure 3. It can be seen from the figure that the probabilities of TN exceedance (S2, S3) were 92.29%, 91.16%, 96.91%, and 88.66% at the DP, GYH, ZL, and DS locations, respectively, indicating that the risk of TN concentration exceedance was high, though the probability of exceedance at the dam site was slightly lower than that at the three river entrance points. The probabilities of TP exceedance (S3), on the other hand, were 18.10%, 8.97%, 24.59%, and 1.31% at the DP, GYH, ZL, and DS locations, respectively. The risk of exceedance was lower at the dam site and significantly higher at the three river entrance points. The risk of exceedance at ZL was higher than that at DP and GYH. The exceedance rates for F.coli (S3) were 7.85%, 12.30%, 1.69%, and 3.94% at the DP, GYH, ZL, and DS locations, respectively. The risks at the DP and GYH locations were higher than at the ZL and DS locations. The exceedance rates for BOD_5_ (S3) at the DP, GYH, ZL, and DS locations were 9.94%, 9.96%, 6.04%, and 1.24%, respectively. As such, it can be seen that the risk of exceedance at the dam site was much lower than that at the entrance. The risk of exceeding the water quality standard was: TN > TP > BOD_5_ > F.coli. According to the pollution source apportionment results, it can be understood that these water quality parameters were influenced by different factors, and demonstrated different characteristics under different conditions of precipitation and runoff.

#### 3.3.2. Water Quality Risk under Different Rainfall Conditions

**Figure 4:**
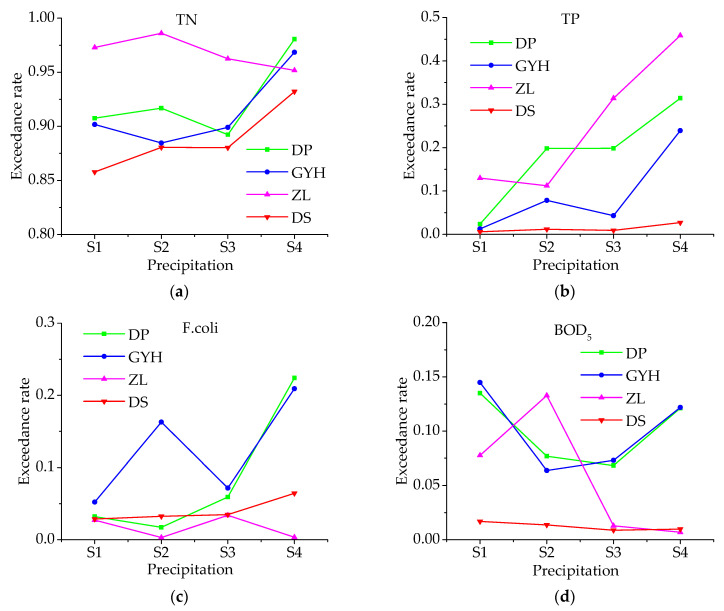
Water quality risk to parameters (**a**) total nitrogen (TN), (**b**) phosphorus (TP), (**c**) fecal coliforms (F.coli), and (**d**) five-day biochemical oxygen demand (BOD5) under different precipitation conditions.

The results on the probability of each water quality condition at the river entrance points and dam site under different precipitation conditions are shown in Table 5 and Figure 4. As can be seen from the table that the probabilities of exceedance for TN, TP, and F.coli increased from 91.00%, 4.30%, and 3.51% to 95.83%, 25.98%, and 12.53% as the monthly rainfall gradually increased from ≤68.25 (S1) to >190.18 (S4). However, the probability of exceedance for BOD_5_ showed a downward and then upward trend. Specifically, the probability of TN exceedance at the dam site increased from 85.79% to 93.21% with the increase in rainfall, and the probability of exceedance at the entrance of DP and GYH increased from 90.75% and 90.17% to 98.06% and 96.85%, respectively, all of which showed a clear upward trend. The exceedance rate of TN at ZL was relatively higher and did not change much with the increase in rainfall. For TP parameters, with the increase in rainfall intensity, the risk of exceeding the water quality standard at the dam site increased from 0.58% to 2.71%, with a slightly increasing trend, while the exceedance rates at the entrance area increased from 2.38%, 1.27% and 12.98% to 31.40%, 23.93%, and 45.89%, with a very obvious increasing trend. For the parameter of F.coli, the exceedances at DP and GYH increased from 3.21% and 5.22% to 22.41% and 20.92% as the rainfall increased, while it at the DS location showed a slight increase. The results indicate that F.coli in the BLH Reservoir mainly comes from the BL River and the GL River. For BOD_5_, the exceedance rate fluctuated mainly at the river entrance points, with little change at the dam site. The risk of BOD_5_ exceedance did not increase with increasing precipitation, indicating that it is less affected by nonpoint source pollution, which is consistent with the results of pollution source apportionment.

#### 3.3.3. The Relationship between Water Quality Risk at the River Entrance Points and Dam Site

**Figure 5:**
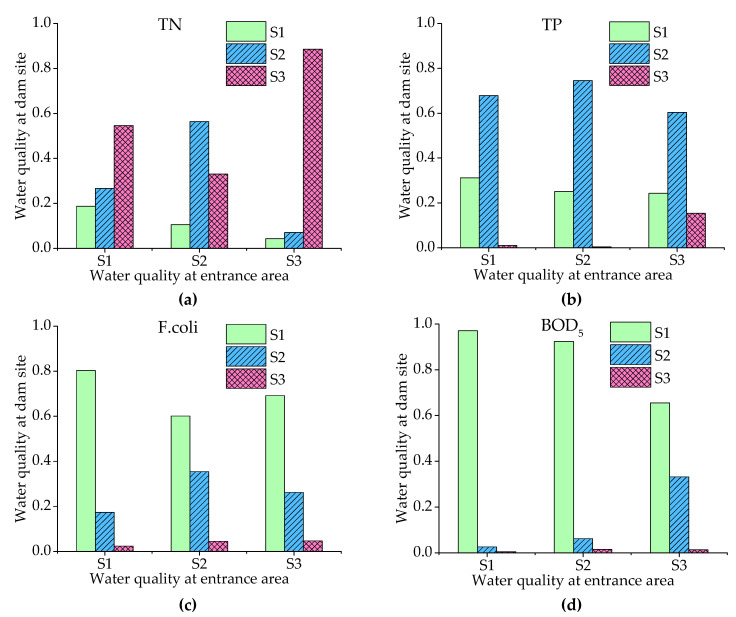
Water quality risk at dam site under different water quality (**a**) total nitrogen (TN), (**b**) phosphorus (TP), (**c**) fecal coliforms (F.coli), and (**d**) five-day biochemical oxygen demand (BOD5) of the entrance area.

The water quality distributions at the dam site under different water quality states at the entrance points are shown in Figure 5. It can be seen from the figure that the risk of the entrance area and dam site were different due to the long distance from the entrance to the dam site. The water quality risk at the dam site was much lower than that in the entrance area. But there was a certain correlation between them. As can be seen from the figure, the probability of exceedance at the dam site gradually increased with the deterioration of water quality at the river entrance points. That is especially true for TN and TP, when the concentrations of the entrance point were in the state of S3, the probabilities of those in the state of S3 were 88.59% and 15.38% at the dam site, which meant that the water quality exceedance rate at the dam site increased greatly with the exceedance of water quality standards at the three river entrance points. As shown in Table 6, Spearman’s rank correlation analysis between the dam site and the river entrance points also showed that water quality at the entrance points significantly affected the water quality at the dam site.

## 4. Discussion

This study mainly analyzed the water quality risk under different rainfall conditions. With the increase of rainfall, there was an increase in the water quality risk of parameters influenced by non-point source pollution. The other hydrological factors included in the Bayesian network had similar characteristics, that is, with the increase of hydrological parameters, the water quality risk tended to increase on the whole. It can be explained that the runoff, water level, and reservoir discharge are directly or indirectly influenced by rainfall. Storm runoff is the main driver of material transport in the watershed area. High flow events carrying large amounts of pollutants into reservoirs are a major cause of water quality degradation during flood seasons. The risk analysis results indicated that TP is strongly influenced by rainfall, and attention should be paid to TP during flood seasons. The correlation analysis demonstrated that TN has a good correlation with runoff and water level, while turbidity has a good correlation with rainfall and runoff (Table 7). The parameters of TP and F.coli, meanwhile, are significantly correlated with turbidity, indicating that TP and F.coli are mainly imported into the reservoir in the form of adsorbed sediment and suspended solids. The correlations between TP, F.coli and hydrological factors were not significant, which indicated the biochemical processes of TP and F.coli are much more complex than turbidity. Besides the hydrological factors, management practices such as fertilization and plant uptake will also result in the non-linear relationship between TP and hydrological factors. The above results confirm that nonpoint source pollution caused by rainfall runoff is the major source of the pollutants in the BLH Reservoir.

**Figure 6:**
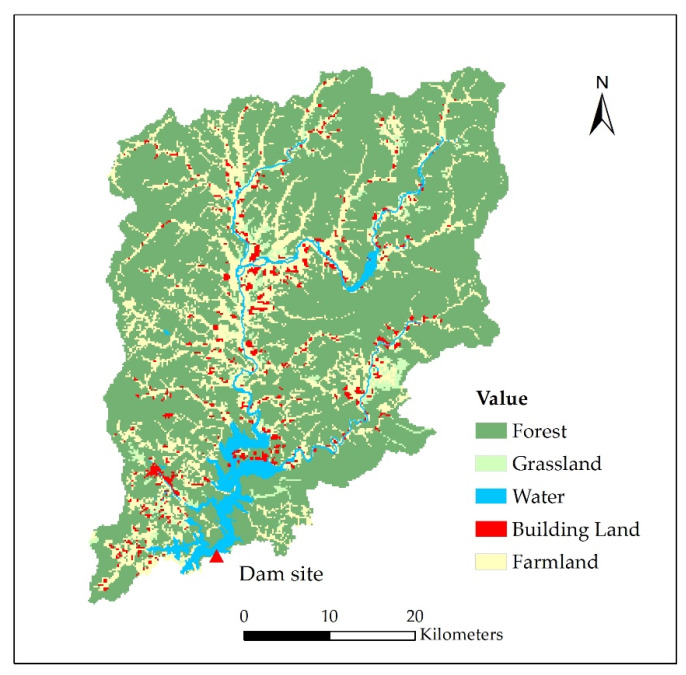
Land use pattern of the BLH (Biliuhe) Reservoir upper watershed.

The land use pattern of the BLH Reservoir upper watershed is shown in Figure 6. The forest and farmland are the main land use types, accounting for 72.3% and 18.9% of the reservoir upper watershed area. Agricultural nonpoint sources are closely related to the land use types of farmland and building land in the watershed area, which corresponding to the agricultural activities and residential sewage discharge. It can be seen that there are a large number of farmland plots and residential areas along the river. When storm runoff occurs, farmland runoff and rural domestic sewage can easily enter the river with the runoff and eventually be transported to the reservoir. Specifically, the total area of farmland and building land accounts for 22.8%, 18.6%, and 33.4% of the watershed area of the BL River, the GL River, and the BJ River, respectively. The large proportion of farmland and building land may lead to serious nutrient loss, which is consistent with the higher risk of TN and TP at the entrance area of ZL. Compared to the BL River and the GL River, the entrance area of the BJ River is relatively closer to the dam site, but the upstream catchment area of the BJ River is much smaller than that of the BL River and the GL River. Therefore, the water quality differed greatly between the ZL and DS. Besides, the BJ River is curved and there are many bays between the entrance area and dam site, which will decrease the influence of the ZL water quality on the DS water quality. To improve the water quality of the reservoir and control the exceedance risk, the nutrients’ loss should be reduced firstly by eradicating excessive fertilization and upgrading traditional agriculture. Second, it is necessary to improve the facilities for livestock and poultry farms and build small sewage treatment plants for the rural areas, which could decrease the fecal contamination effectively. In addition, large amounts of floating debris could enter the reservoir during flood periods, which require timely treatment. Besides this, the implementation of an artificial wetland in the reservoir buffer zone presents an effective measure for intercepting the pollutants in the residential areas around the reservoir, promoting the degradation of the pollutants before they enter the reservoir, and preventing the threat of sudden water pollution events. The water quality is dynamic during flood seasons. In general, the water quality is poor at the beginning of the flood due to the eroded pollutants from the watershed, which then has a dilution effect in the post-period of the flood [17]. Discharging runoff with higher pollution concentrations and storing incoming flows with better water quality through reasonable regulation measures can alleviate the water quality risks during flood periods. The water quality risk to the BLH Reservoir can be decreased through comprehensive measures of watershed management practices, entrance interception, and reservoir regulation.

The proposed research framework in this paper, including water quality analysis, pollution source identification, risk assessment, and water quality risk control, can be applied to protect the water quality of reservoir water sources and, thus, ensure the safety of the urban water supply. In the construction of the Bayesian network, the relationship between hydrological parameters and water quality parameters is simplified, which will cause a certain amount of error and uncertainty. A more accurate and specific description of the structure about hydrological and water quality factors should be constructed to reduce the uncertainty of the model. Besides, Bayesian networks can use the posterior data to continuously improve the accuracy of the model. In the future, it is necessary to increase the frequency of water quality sampling under special weather conditions such as floods. For the BLH Reservoir basin, storm runoff is the main driving factor of the pollutants transportation. Existing research has shown that the frequency of extreme rainfall is increasing due to climate change, which means that the risk to water quality as a result of storm runoff is increasing [49,50,51]. The changes to water quality risk induced by climate change should be evaluated further to provide a basis for future water resource management.

## 5. Conclusions

In this paper, the water quality characteristics of a drinking water reservoir during flood seasons were selected for analysis, the main pollution sources were identified by the PCA/FA-MLR model, and the water quality risk was evaluated by the Bayesian networks model, then the management strategies were proposed to alleviate the water quality risk in the watershed. The main conclusions are summarized as follows:

(1) General water quality data for the BLH Reservoir were analyzed to identify the water quality parameters that exceeded the standard. The results showed that TN, TP, F.coli, and BOD_5_ were the key risk factors during flood seasons.
(2) Based on the PCA/FA-MLR receptor model, it was found that agricultural nonpoint source pollution has the greatest impact on the water quality of the BLH Reservoir during flood seasons, contributing 53.20%, 48.41%, 72.69%, and 68.06% of the total nitrogen, phosphorus, fecal coliforms, and turbidity, respectively.
(3) A Bayesian network model was employed to assess the risk to water quality during flood seasons, and the results showed that the risk of water quality exceedances gradually increased with the increase of rainfall. The probability of exceedance for TN, TP, and F.coli increased from 91.00%, 4.30%, and 3.51% to 95.83%, 25.98%, and 12.53% as the monthly rainfall increased from ≤68.25 to >190.18. The risk of BOD_5_ exceeding the standard, however, did not increase with the increase in rainfall. The risk of exceedance of water quality standards at the entrance points was greater than that at the dam site.
(4) Agricultural nonpoint source pollution driven by storm runoff is a major risk factor for reservoir water quality and should be addressed as a priority. The proposed research framework of water quality analysis, pollution source identification, risk assessment, and water quality risk control can be applied to protect the water quality of reservoir water sources and ensure the safety of the urban water supply.

## Figures and Tables

**Table 1:** Water quality standard of surface water in China (GB3838–2002) mg/L.

Parameters	National Surface Water Quality Standard (GB3838-2002)				
	I	II	III	IV	V
pH	6~9			<6 or >9	
DO (mg/L)	≥7.5	6.0	5.0	3.0	2.0
COD_Mn_ (mg/L)	≤2.0	4.0	6.0	10.0	15.0
BOD_5_ (mg/L)	≤3.0	3.0	4.0	6.0	10.0
NH_3_-N (mg/L)	≤0.15	0.5	1.0	1.5	2.0
TN (mg/L)	≤0.2	0.5	1.0	1.5	2.0
TP (mg/L)	≤0.01	0.025	0.05	0.10	0.20
F.coli (A/L)	≤200	2000	10,000	20,000	40,000
F^−^ (mg/L)	≤1.0	1.0	1.0	1.5	1.5
Cl^−^ (mg/L)	≤250			>250	
NO_3_-N (mg/L)	≤10			>10	
Turbidity (NTU)	-				

**Table 2:** General water quality characteristics of the BLH Reservoir ^1^.

Parameters	Non-Flood Seasons				Flood Seasons			
	Mean	SD	Min	Max	Mean	SD	Min	Max
pH	7.8	0.3	7.0	8.5	8.0	0.4	7.1	9.0
DO	10.41	1.84	6.20	14.20	7.56	1.33	5.16	11.70
COD_Mn_	2.22	0.48	1.10	3.30	2.51	0.50	1.58	4.41
BOD_5_	1.95	0.84	0.52	4.51	1.72	0.77	0.35	4.15
NH_3_-N	0.17	0.14	0.01	0.61	0.15	0.13	0.01	0.66
NO_3_^−^-N	1.62	0.93	0.10	3.68	1.45	0.90	0.10	3.26
TN	2.45	0.95	0.38	4.83	2.34	1.08	0.28	5.14
TP	0.018	0.009	0.004	0.051	0.021	0.013	0.001	0.097
F.coli	84	126	2	841	875	2392	2	16,165
TUB	2.9	5.3	0.1	37.6	5.5	6.4	0.4	34.3
F^−^	0.20	0.05	0.08	0.34	0.19	0.04	0.04	0.29
Cl^−^	10.82	1.40	6.71	13.23	10.88	2.58	6.56	29.20

^1^ pH, -; F.coli, A/L; TUB, NTU; others, mg/L.

**Table 3:** Water quality index load in flood seasons.

Parameters	Factor 1	Factor 2	Factor 3	Factor 4	Factor Commonality
pH	0.065	0.088	0.097	0.859	0.765
DO	0.019	0.103	0.067	0.865	0.744
COD_Mn_	0.319	0.778	−0.083	−0.083	0.732
BOD_5_	0.006	0.802	0.108	0.415	0.812
NH_3_-N	0.130	0.492	0.748	0.042	0.752
NO_3_-N	0.582	0.180	0.570	0.156	0.711
TN	0.646	0.077	0.447	−0.044	0.668
TP	0.625	0.394	0.189	−0.084	0.578
F.coli	0.877	0.131	0.134	0.064	0.854
TUB	0.903	0.232	0.079	0.113	0.910
F^−^	0.382	0.704	0.184	0.153	0.719
Cl^−^	0.214	−0.142	0.784	0.139	0.638
Initial Eigenvalue	4.859	1.771	1.370	0.918	
% of Variance	25.385	19.068	15.210	14.656	
Cumulative %	25.385	44.453	59.663	74.319	

**Table 4:** Source contributions to concentrations of water quality parameters (%) ^1^.

Parameters	Source 1	Source 2	Source 3	Source 4	Adjusted R^2^
pH	5.86	7.97	8.77	77.41	0.73
DO	1.77	9.81	6.32	82.10	0.74
COD_Mn_	25.25	61.59	6.57	6.59	0.69
BOD_5_	0.43	60.28	8.13	31.16	0.81
NH_3_-N	9.20	34.83	52.98	3.00	0.80
NO_3_-N	39.12	12.12	38.27	10.49	0.69
TN	53.20	6.38	36.83	3.59	0.59
TP	48.41	30.50	14.60	6.50	0.55
F.coli	72.69	10.86	11.13	5.31	0.79
TUB	68.06	17.45	5.99	8.51	0.88
F^−^	26.86	49.45	12.90	10.78	0.67
Cl^−^	16.71	11.11	61.31	10.86	0.67

^1^ Source 1, agricultural nonpoint source of pollution driven by rainfall and runoff; Source 2, rural and urban sewage discharge source; Source 3, groundwater pollution source; Source 4, meteorological factors.

**Table 5:** Risk of the reservoir water quality under different precipitation conditions.

Precipitation (mm)	≤68.25	68.25–119.46	119.46–190.18	>190.18
TN	91.13%	91.65%	90.86%	95.75%
TP	3.40%	9.82%	14.13%	25.77%
F.coli	3.34%	5.46%	4.96%	12.76%
BOD_5_	9.28%	7.30%	4.21%	6.53%

**Table 6:** Spearman’s rank correlation coefficients between the dam site and the river entrance points.

r	DS			
	TN	TP	F.coli	BOD_5_
DP	0.830 **	0.580 **	0.577 **	0.618 **
GYH	0.890 **	0.602 **	0.622 **	0.663 **
ZL	0.834 **	0.468 **	0.206 *	0.687 **

**, *p* < 0.01; *, *p* < 0.05.

**Table 7:** Spearman’s rank correlation coefficients between hydrological factors and the major water quality parameters.

r	P	R	W	BOD_5_	TN	TP	F.coli	TUB
P	1.000							
R	0.701 **	1.000						
W	0.012	0.242 **	1.000					
BOD_5_	−0.059	−0.177 *	−0.351 **	1.000				
TN	0.062	0.391 **	0.448 **	0.020	1.000			
TP	0.105	0.145	−0.118	0.255 **	0.154	1.000		
F.coli	−0.119	−0.131	−0.246	0.068	−0.183	0.247	1.000	
TUB	0.548 **	0.552 **	0.042	0.209 *	0.439 **	0.488 **	0.677 **	1.000

**, *p* <0.01; *, *p* <0.05.

## Data Availability

No new data were created or analyzed in this study. Data sharing is not applicable to this article.

## Appendix A

**Table A1:** Standard attribute table of hydrological factors’ discretization.

Hydrological Factors	Attribute Values			
	S1	S2	S3	S4
Precipitation (mm)	≤68.25	68.25–119.46	119.46–190.18	>190.18
Runoff (10^6^ m^3^)	≤14.14	14.14–66.74	66.74–175.92	>175.92
Water level (m)	≤59.47	59.47–63.24	63.24–67.12	>67.12
Discharge (10^6^ m^3^)	≤15.93	15.93–29.43	29.43–41.64	>41.64

**Table A2:** Standard attribute table of water quality parameters’ discretization.

Water Quality Parameters	Attribute Values		
	S1	S2	S3
TN (mg/L)	≤1	1.0–2.0	>2.0
TP (mg/L)	≤0.01	0.01–0.05	>0.05
F.coli (A/L)	≤200	200–10,000	>10,000
BOD_5_ (mg/L)	≤3	3–4	>4

## References

[1] Oki T. (2006). Global Hydrological Cycles and World Water Resources. Science.

[2] Zhou Y., Khu S., Xi B., Sun J., Hao F., Wu J., Huo S. (2014). Status and challenges of water pollution problems in China: Learning from the European experience. Environ. Earth Sci..

[3] Tzanakakis V.A., Paranychianakis N.V., Angelakis A.N. (2020). Water Supply and Water Scarcity. Water.

[4] Azadi F., Ashofteh P., Loáiciga H.A. (2018). Reservoir Water-Quality Projections under Climate-Change Conditions. Water Resour. Manag..

[5] Xu Z., Cai X., Yin X., Su M., Wu Y., Yang Z. (2019). Is water shortage risk decreased at the expense of deteriorating water quality in a large water supply reservoir?. Water Res..

[6] Zhang C., Gao X., Wang L., Chen Y. (2013). Analysis of agricultural pollution by flood flow impact on water quality in a reservoir using a three-dimensional water quality model. J. Hydroinform..

[7] Delpla I., Jung A.V., Baures E., Clement M., Thomas O. (2009). Impacts of climate change on surface water quality in relation to drinking water production. Environ. Int..

[8] Ma W., Huang T., Li X., Zhou Z., Li Y., Zeng K. (2015). The Effects of Storm Runoff on Water Quality and the Coping Strategy of a Deep Canyon-Shaped Source Water Reservoir in China. Int. J. Env. Res. Public Health.

[9] de Man H., van den Berg H.H.J.L., Leenen E.J.T.M., Schijven J.F., Schets F.M., van der Vlient J.C., van Knapen F., Husman A.M.D. (2014). Quantitative assessment of infection risk from exposure to waterborne pathogens in urban floodwater. Water Res..

[10] Barber L.B., Paschke S.S., Battaglin W.A., Douville C., Fitzgerald K.C., Keefe S.H., Roth D.A., Vajda A.M. (2017). Effects of an Extreme Flood on Trace Elements in River Water From Urban Stream to Major River Basin. Environ. Sci. Technol..

[11] Rui Y., Fu D., Do Minh H., Radhakrishnan M., Zevenbergen C., Pathirana A. (2018). Urban Surface Water Quality, Flood Water Quality and Human Health Impacts in Chinese Cities. What Do We Know?. Water.

[12] St-Hilaire A., Duchesne S., Rousseau A.N. (2016). Floods and water quality in Canada: A review of the interactions with urbanization, agriculture and forestry. Can. Water Resour. J..

[13] Curriero F.C., Patz J.A., Rose J.B., Lele S. (2001). The association between extreme precipitation and waterborne disease outbreaks in the United States, 1948-1994. Am. J. Public Health.

[14] Zhou F., Huang G.H., Guo H., Zhang W., Hao Z. (2007). Spatio-temporal patterns and source apportionment of coastal water pollution in eastern Hong Kong. Water Res..

[15] Gholizadeh M.H., Melesse A.M., Reddi L. (2016). Water quality assessment and apportionment of pollution sources using APCS-MLR and PMF receptor modeling techniques in three major rivers of South Florida. Sci. Total Environ..

[16] Qin G., Liu J., Xu S., Wang T. (2020). Water quality assessment and pollution source apportionment in a highly regulated river of Northeast China. Environ. Monit. Assess..

[17] Baborowski M., Simeonov V., Einax J.W. (2012). Assessment of Water Quality in the Elbe River at Flood Water Conditions Based on Cluster Analysis, Principle Components Analysis, and Source Apportionment. CLEAN-Soil Air Water.

[18] Ota Y., Suzuki A., Yamaoka K., Nagao M., Tanaka Y., Irizuki T., Fujiwara O., Yoshioka K., Kawagata S., Kawano S. (2021). Geochemical distribution of heavy metal elements and potential ecological risk assessment of Matsushima Bay sediments during 2012–2016. Sci. Total Environ..

[19] Liu R., Men C., Yu W., Xu F., Wang Q., Shen Z. (2018). Uncertainty in positive matrix factorization solutions for PAHs in surface sediments of the Yangtze River Estuary in different seasons. Chemosphere.

[20] Wang Z., Wang T., Liu X., Hu S., Ma L., Sun X. (2020). Water Level Decline in a Reservoir: Implications for Water Quality Variation and Pollution Source Identification. Int. J. Environ. Res. Public Health.

[21] Li C., Li S.L., Yue F.J., Liu J., Zhong J., Yan Z., Zhang R., Wang Z., Xu S. (2019). Identification of sources and transformations of nitrate in the Xijiang River using nitrate isotopes and Bayesian model. Sci. Total Environ..

[22] Jin Z., Qin X., Chen L., Jin M., Li F. (2015). Using dual isotopes to evaluate sources and transformations of nitrate in the West Lake watershed, eastern China. J. Contam. Hydrol..

[23] Yang X., Liu Q., Fu G., He Y., Luo X., Zheng Z. (2016). Spatiotemporal patterns and source attribution of nitrogen load in a river basin with complex pollution sources. Water Res..

[24] Wang Y., He B., Duan W., Li W., Luo P., Razafindrabe B.H.N. (2016). Source Apportionment of Annual Water Pollution Loads in River Basins by Remote-Sensed Land Cover Classification. Water.

[25] Abdullahi K.L., Delgado-Saborit J.M., Harrison R.M. (2018). Sensitivity of a Chemical Mass Balance model for PM2.5 to source profiles for differing styles of cooking. Atmos. Environ..

[26] Wu Y., Chen J. (2013). Investigating the effects of point source and nonpoint source pollution on the water quality of the East River (Dongjiang) in South China. Ecol. Indic..

[27] Larson T., Gould T., Riley E.A., Austin E., Fintzi J., Sheppard L., Yost M., Simpson C. (2017). Ambient Air Quality Measurements from a Continuously Moving Mobile Platform: Estimation of Area-Wide, Fuel-Based, Mobile Source Emission Factors Using Absolute Principal Component Scores. Atmos. Environ..

[28] Huang F., Wang X., Lou L., Zhou Z., Wu J. (2010). Spatial variation and source apportionment of water pollution in Qiantang River (China) using statistical techniques. Water Res..

[29] Dou M., Zhang Y., Zuo Q., Mi Q. (2015). Identification of key factors affecting the water pollutant concentration in the sluice-controlled river reaches of the Shaying River in China via statistical analysis methods. Environ. Sci. Process Impacts.

[30] Chen L., Yang Z., Liu H. (2016). Assessing the eutrophication risk of the Danjiangkou Reservoir based on the EFDC model. Ecol. Eng..

[31] Bjorklund K., Bondelind M., Karlsson A., Karlsson D., Sokolova E. (2018). Hydrodynamic modelling of the influence of stormwater and combined sewer overflows on receiving water quality: Benzo(a)pyrene and copper risks to recreational water. J. Environ. Manag..

[32] Wang J., Yan H., Xin K., Tao T. (2020). Risk assessment methodology for iron stability under water quality factors based on fuzzy comprehensive evaluation. Environ. Sci. Eur..

[33] Liu Y.G., Yang Y.X., Wang L.G. (2015). River Water Quality Risk Assessment Using Grey Correlation in Baoji Weihe River of China. Appl. Mech. Mater..

[34] Wellen C., Arhonditsis G.B., Long T., Boyd D. (2014). Accommodating environmental thresholds and extreme events in hydrological models: A Bayesian approach. J. Great Lakes Res..

[35] Pérez-Miñana E. (2016). Improving ecosystem services modelling: Insights from a Bayesian network tools review. Environ. Model. Softw..

[36] Aguilera P.A., Fernández A., Fernández R., Rumi R., Salmeron A. (2011). Bayesian networks in environmental modelling. Environ. Model. Softw..

[37] Bertone E., Sahin O., Richards R., Roiko R.A. Bayesian Network and system thinking modelling to manage water-related health risks from extreme events. Proceedings of the IEEE International Conference onIndustrial Engineering and Engineering Management (IEEM).

[38] Liang Z., Soranno P.A., Wagner T. (2020). The role of phosphorus and nitrogen on chlorophyll a: Evidence from hundreds of lakes. Water Res..

[39] Goulding R., Jayasuriya N., Horan E. (2012). A Bayesian network model to assess the public health risk associated with wet weather sewer overflows discharging into waterways. Water Res..

[40] Tang C., Yi Y., Yang Z., Sun J. (2016). Risk forecasting of pollution accidents based on an integrated Bayesian Network and water quality model for the South to North Water Transfer Project. Ecol. Eng..

[41] Duan W., He B., Nover D., Yang G., Chen W., Meng H., Zou S., Liu C. (2016). Water Quality Assessment and Pollution Source Identification of the Eastern Poyang Lake Basin Using Multivariate Statistical Methods. Sustainability.

[42] Shehzad M.T., Murtaza G., Shafeeque M., Sabir M., Nawaz H., Khan M.J. (2019). Assessment of trace elements in urban topsoils of Rawalpindi-Pakistan: A principal component analysis approach. Environ. Monit. Assess..

[43] Thurston G.D., Spengler J.D. (1987). A quantitative assessment of source contributions to inhalable particulate matter pollution in metropolitan Boston. Atmos. Environ..

[44] Wijesiri B., Egodawatta P., McGree J., Goonetilleke A. (2016). Assessing uncertainty in stormwater quality modelling. Water Res..

[45] Marcot B.G., Steventon J. D., Sutherland G.D., McCann R.K. (2006). Guidelines for developing and updating Bayesian belief networks applied to ecological modeling and conservation. Can. J. For. Res..

[46] Chen S.H., Pollino C.A. (2012). Good practice in Bayesian network modelling. Environ. Model. Softw..

[47] Ma X., Xing Y., Lu J. (2018). Causation Analysis of Hazardous Material Road Transportation Accidents by Bayesian Network Using Genie. J. Adv. Transp..

[48] Wang T., Xu S., Liu J. (2018). Analysis of accumulation formation of sediment contamination in reservoirs after decades of running: A case study of nitrogen accumulation in Biliuhe Reservoir. Environ. Sci. Pollut. Res..

[49] Westra S., Fowler H.J., Evans J.P., Alexander L.V., Berg P., Johnson F., Kendon E.J., Lenderink G., Roberts N.M. (2014). Future changes to the intensity and frequency of short-duration extreme rainfall. Rev. Geophys..

[50] Arnell N.W., Gosling S.N. (2016). The impacts of climate change on river flood risk at the global scale. Clim. Change.

[51] Lyubimova T., Lepikhin A., Parshakova Y., Tiunov A. (2016). The risk of river pollution due to washout from contaminated floodplain water bodies during periods of high magnitude floods. J. Hydrol..

